# Medicine expenses and obesity in Brazil: an analysis based on the household budget survey

**DOI:** 10.1186/s12889-016-2709-6

**Published:** 2016-01-20

**Authors:** Daniela S. Canella, Hillegonda M. D. Novaes, Renata B. Levy

**Affiliations:** 1 Programa de Pós-Graduação em Nutrição em Saúde Pública, Faculdade de Saúde Pública, Universidade de São Paulo, Avenida Doutor Arnaldo, 715, 01246-904 São Paulo, SP Brazil; 2 Departamento de Medicina Preventiva, Faculdade de Medicina, Universidade de São Paulo, Avenida Doutor Arnaldo, 455, 01246-903 São Paulo, SP Brazil

**Keywords:** Costs, Obesity, Pharmaceuticals, Health systems, Household budget survey, Public health, Epidemiology

## Abstract

**Background:**

Obesity can be considered a global public health problem that affects virtually all countries worldwide and results in greater use of healthcare services and higher healthcare costs. We aimed to describe average monthly household medicine expenses according to source of funding, public or private, and to estimate the influence of the presence of obese residents in households on total medicine expenses.

**Methods:**

This study was based on data from the 2008–2009 Brazilian Household Budget Survey, with a representative population sample of 55,970 households as study units. Information on nutritional status and medicines acquired and their cost in the past 30 days were analyzed. A two-part model was employed to assess the influence of obesity on medicine expenses, with monthly household medicine expenses per capita as outcome, presence of obese in the household as explanatory variable, and adjustment for confounding variables.

**Results:**

Out-of-pocket expenses on medicines were always higher than the cost of medicines obtained through the public sector, and 32 % of households had at least one obese as resident. Monthly household expenses on medicines per capita in households with obese was US$ 20.40, 16 % higher than in households with no obese. An adjusted model confirmed that the presence of obese in the households increased medicine expenses.

**Conclusion:**

Obesity is associated with additional medicine expenses, increasing the negative impact on household budgets and public expenditure.

## Background

Obesity can be considered a global public health problem that affects virtually all countries worldwide, including both developing and developed nations [1]. According to global estimates, between 1980 and 2008, obesity rates rose from 7.9 to 13.8 % among women and from 4.8 to 9.8 % in men [2]. In Brazil, obesity is increasing, according to national surveys conducted since the 1970s, across all age groups and genders [3]. In the adult population of state capitals, obesity has affected an additional 1 % of the population every year, since the mid-2000s [4].

Obesity, and the higher risk for several associated non-communicable diseases (NCDs), results in greater use of healthcare services and higher healthcare costs [5]. Since the 1990s, researchers in developed countries have analysed the cost of obesity and recognized its significant economic impact on health systems [6–9]. In Brazil, there are few data about this topic and all refer to the perspective of society. The first study investigated the burden of hospitalization due to obesity in 2001 [10] and later two studies evaluated the direct costs associated to outpatient and inpatient care of obesity based on data from public health information systems (2008–2011). These studies showed the high cost of overweight and/or obesity for the Brazilian national health system (Sistema Único de Saúde - SUS) [11, 12] used by 75 % of the population [13, 14].

These studies, however, did not present data on medicine expenses. The available databases provide limited information on the utilization and cost of these products in the public health system [12] and private expenses, corresponding to out-of-pocket payments by the population, are not included at all. Given the current gaps in knowledge, and the availability of data regarding expenses on medicines in the Brazilian Household Budget Survey, the objective of the present study was to describe average monthly per capita household expenses on medicines and to estimate the influence of the presence of obese residents in households on total household expenses on medicines.

## Methods

### Data source and sample

The present study used data of the Household Budget Survey (HBS) conducted by the Brazilian Institute of Geography and Statistics (IBGE) between May 2008 and May 2009.

This was a population-based study involving 55,970 Brazilian households, based on a complex sample plan using two-stage cluster sampling, with random selection of census sectors during the first stage and of households in the second stage. For selection of census sectors, the 12,800 sectors present in the Master Sample of Household Surveys were grouped to obtain household strata with high geographic and socioeconomic homogeneity. The geographic location of the sector (region, state, and urban or rural area), as well as internally in each geographic locus, and the spectrum of socioeconomic variation of the families (based on the income of head of household), were considered for this grouping. The number of households randomly selected from each stratum was proportional to the total number of households in the stratum. Interviews with households in each stratum were distributed uniformly over the four quarters of the year in order to represent the seasonal variations of the expenditures [15].

### Data collection

The information from the HBS relevant to the objectives of this study were medicine expenses (monetary or non-monetary), nutritional status of household residents and sociodemographic data.

Medicine expenses were registered in a specific instrument, to be answered by residents aged 10 years or older, and include medicine expenses of all members of the household. All medicines referred in the survey were included in the study.

The reference period for expenses on medicines was the 30 days previous to the interview. Information was collected for acquisition modes (monetary or non-monetary), cost, place of acquisition and characteristics of the product (brand name, generic, plant and herbal medicine, compounded, or homeopathic).

Regarding non-monetary expenses, i.e. medicines distributed without charge by the health system, their cost was estimated by the residents based on local market prices [15].

All individuals residing in the household were included in the nutritional status assessment, excluding pregnant women (*n* = 188,461). Weights and heights were measured using standard techniques by the researcher and recorded in specific questionnaires, along with characteristics of the residents and of the household. Weight was measured using portable electronic scales with a maximum capacity of 150(kg), and graduations of 100(g). The value obtained was recorded in kilograms. Height was expressed in centimetres (cm) using length as the measure in children aged between zero and 23 months and stature in individuals aged 24 months or older. In order to measure length, infant anthropometres were used with a capacity of up to 105 cm and a scale in millimetres, whereas stature was measured using portable stadiometres with a 200 cm-long retractable tape measure, accurate to the nearest 0.1 cm [3].

### Assessment of spending on medicines

Although medicine expenses are related to individual needs, the household was the study unit, considering that these expenses rarely depend only on an individual decision [16] but are related to family dynamics [17] and only individuals aged 10 years or older reported expenses, and the children’s expenses were reported by other members of the household.

Total spending on medicines was divided in expenses in the public sector (obtained in the SUS and in pharmaceutical assistance programs) and in the private sector (paid for out-of-pocket). To this end, information of product acquisition (monetary or non-monetary) and place of obtainment were used. The deflated amount of the spending was calculated, using as reference January 15th, 2009 and the Extended Consumer Price Index (Índice Nacional de Preços ao Consumidor Amplo-IPCA), in order to correct the absolute and relative shifts in prices during the year [15]. Expenses in Reals were converted into US dollars using a purchasing power parity basis (PPP 2009: US$ 1.00 = RS 1.63) [18].

Household expenses on medicines were summed up and divided by the number of residents, obtaining monthly per capita household medicine expenses.

### Assessment of nutritional status

Based on weight and height measurements taken, body mass index (BMI) was calculated for adults and elderly, whereas BMI-for-age was calculated for children and adolescents and expressed as z-score. The nutritional status of individuals was classified according to recommendations by the World Health Organization (WHO) for each age group. Obesity was defined as a BMI of above 30 kg/m^2^ for adults and elderly, +3 z-scores for children under 5 years of age, and +2 z-scores for children and adolescents (5–19 years) [19–21].

Considering the households as study units, the proportion of households containing at least one obese resident (presence of obese residents) was calculated.

### Data analysis

Sociodemographic characteristics were expressed per quintiles of per capita household income. A descriptive analysis of monthly per capita household expenses on medicines was performed, for the public and private sector, and the proportion of households with at least one obese resident, according to sociodemographic variables. Monthly per capita household expenses on medicines, according to source of funding and total spending, were described according to the presence of obese in the households. Households with and without obese were compared with the difference of means tests.

The association between the presence of obese in household and monthly per capita expenses on medicines was analysed using two-part model (TPM), a recommended method in the analysis of cost of obesity data, as it takes into account that a proportion of the population does not have health expenditures at all [22–25]. In our case, 17 % of households did not incur any expenses in the 30 days before the survey. According to this method one equation predicts the probability that a person has any expenditure, using logistic regression, and a second equation predicts the level of expenditure, using linear regression [23, 24]. Both equations were adjusted for social and family characteristics that influence the presence of obese residents and expenses on medicines in households such as: proportion of children aged under 2 years, children aged 2 to 9, adolescents, elderly and of women in the household, presence of pregnant women in the household, region, area, and monthly household income per capita. The predicted coefficient value, considering the coefficients of the two equations, was calculated.

All analyses were carried out using the statistics package Stata/SE version 12.1 (Stata Corp., College Station, USA) in the survey module, which considers the effects of complex sampling and enables the extrapolation of results for the Brazilian population, considering a 95 % confidence interval and 5 % significance level.

### Ethical aspects

The study was approved by the Research Ethics Committee of School of Public Health, University of São Paulo (Process number 2292). This study utilized data from the HBS 2008–2009, collected by the IBGE, a public database available online. The information contained in the database is confidential since specific data about each household such as identification of the household members, address and telephone are excluded.

## Results

A total of 55,970 Brazilian households were studied. Of this total, 84.4 % were situated in the urban area and 67.1 % in the Mid-South of the country. The households had an average of 3.3 residents, 3.2 % of whom were children under 2 years, 8.9 % children aged 2–9 years, 14.1 % adolescents, 11.6 % elderly, and 51.8 % women. Average monthly household income per capita was US$ 645 and 3.4 % of the income was dedicated to cover monthly private expenses on medicines (out-of-pocket) (Table 1).

**Table 1:** Household distribution of sociodemographic characteristics, by quintiles of household income. Brazil, 2008–2009

Variables	Quintiles of household income per capita (mean values)					Brazil
	1st	2nd	3rd	4th	5th	
Area (%)						
Rural	31.1	18.5	14.6	9.0	4.7	15.6
Urban	68.9	81.5	85.4	91.0	95.3	84.4
Region (%)						
North and Northeast	61.4	40.2	28.3	19.0	15.8	33.0
Mid-South	38.6	59.8	71.7	81.0	84.2	67.1
Number of dwellers in households	4.5	3.7	3.0	2.8	2.5	3.3
Proportion of children under 2 years of age in family unit (%)	6.4	4.0	2.5	1.9	1.4	3.2
Proportion of children 2–9 years of age in family unit (%)	17.2	11.2	6.9	5.6	3.7	8.9
Proportion of adolescents (10–19 years) in family unit (%)	23.0	18.1	12.3	9.7	7.5	14.1
Proportion of elderly (65 years or older) in family unit (%)	2.6	7.1	16.8	15.9	15.7	11.6
Proportion of women in family unit (%)	50.7	51.3	51.6	52.7	52.6	51.8
Monthly household income per capita (US$)	97.22	213.22	353.00	591.38	1968.82	644.70
Proportion of monthly household income per capita dedicated to monthly medicine expenses per capita (out-of-pocket expenditure) (%)	5.1	3.6	3.3	2.9	2.0	3.4

Considering all households, 17 % (*n* = 9,730) did not have any expenses on medicines in the last month (data not shown). Out-of-pocket expenses on medicines were higher than for those obtained through the public sector, in urban and rural areas, all regions and income groups. Out-of-pocket expenses exceeded public expenses even in the 20 % poorest stratum of the population (Table 2).

**Table 2:** Distribution of medicine expenses, according to public and private sector and sociodemographic variables. Brazil, 2008–2009

Variables	Monthly household medicine expenses per capita^a^				
	Public Sector (obtained in the SUS)		Private Sector (paid for out-of-pocket)		Total
	US$	% spent on medicines	US$	% spent on medicines	US$
Area					
Rural	2.32	16.13	9.39	83.87	11.71
Urban	3.32	12.01	16.41	87.99	19.73
Regions					
North and Northeast	1.71	11.42	9.31	88.58	11.01
Mid-South	3.88	13.23	18.26	86.77	22.14
Household income per capita (US$)					
1st quintile	1.01	16.37	4.34	83.63	5.35
2nd quintile	2.13	15.36	8.04	84.64	10.17
3rd quintile	3.46	15.33	12.18	84.67	15.64
4th quintile	4.08	11.51	18.16	88.49	22.24
5th quintile	5.17	5.11	33.82	94.89	38.99
Brazil	3.17	12.64	15.31	87.36	18.48

^a^Values in dollars (US$)

Of all the households studied, 32.1 % had at least one obese individual in the household, and this percentage was higher in the urban area, in the Mid-South of the country and in the intermediate income levels (Table 3). In the Brazilian population, considering all age groups, the prevalence of obesity was 13.2 % (data not shown).

**Table 3:** Distribution of proportion of households with obese individuals, according to sociodemographic variables. Brazil, 2008–2009

Variables	Proportion of households with at least one obese individual (%) (presence of obese in household)^a,b^
Area	
Rural	28.63
Urban	32.75
Regions	
North and Northeast	29.42
Mid-South	33.43
Household income per capita (US$)	
1st quintile	30.36
2nd quintile	32.14
3rd quintile	32.67
4th quintile	33.48
5th quintile	31.89
Brazil	32.11

^a^Includes individuals of all age groups and excluding pregnant women

^b^Classification according to recommendations by the World Health Organization for each age group: BMI above 30 kg/m^2^, for adults and elderly, +3 z-scores, for children under 5 years, and +2 z-scores, for children and adolescents (5–19 years)

Average monthly per capita household medicine expenses in households with obese residents were US$20.40, 16 % higher than expenses in households with no obese individuals. Out-of-pocket expenses on medicines were 11 % higher in households with at least one obese (Table 4).

**Table 4:** Average of medicine expenses, according to the presence of obese in households. Brazil, 2008–2009

Presence of obese in household^a,b^	Monthly household medicine expenses per capita^c^		
	Public Sector (obtained in the SUS)	Private Sector (paid for out-of-pocket)	Total medicine expenses
No	2.77	14.80	17.57
Yes	4.01	16.39^*^	20.40^*^

^*^p < 0.05

^a^Includes individuals of all age groups and excluding pregnant women

^b^Classification according to recommendations by the World Health Organization for each age group: BMI above 30 kg/m^2^, for adults and elderly, +3 z-scores, for children under 5 years, and +2 z-scores, for children and adolescents (5–19 years)

^c^Values in dollars (US$)

Adjusted and predicted coefficients showed a statistically significant positive association for higher expenses on medicines attributable to the presence of obese residents in the household. After adjusting for confounding variables, results showed that the presence of obese in the households was associated with 19 % higher monthly expenses on medicines per capita compared to households with no obese residents (Table 5).

**Table 5:** Association between medicine expenses and presence of obese in households. Brazil, 2008–2009

Variables	Monthly household per capita medicine expenses		
	Adjusted analyses		
	Logistic regression^c^	Linear regression^d^	Predicted (TPM combined)
	Coefficient	Coefficient	Coefficient
	[95 % CI]	[95 % CI]	[95 % CI]
Presence of obese in household^a,b^ (0 = no; 1 = yes)	0.29	3.15	3.35
	[0.21; 0.37]	[1.03; 5.28]	[1.54; 5.15]
Proportion of elderly (65 years or older)	0.84	32.30	29.26
	[0.66; 1.02]	[28.21; 36.40]	[25.78; 32.73]
Proportion of children under 2 years	1.71	−14.30	−7.96
	[1.29; 2.12]	[−18.38;−10.23]	[−11.51;−4.41]
Proportion of children (2–9 years)	0.37	−22.31	−17.93
	[0.13; 0.61]	[−25.51;−18.87]	[−20.87;−14.98]
Proportion of adolescents (10–19 years)	0.26	−17.87	−14.45
	[0.07; 0.45]	[−25.74;−18.87]	[−16.99;−11.92]
Proportion of women	0.95	11.82	12.25
	[0.78; 1.13]	[8.45; 15.19]	[9.36; 15.14]
Presence of pregnant women	0.12	3.18	2.96
	[−0.08; 0.32]	[−6.11; 12.48]	[−4.90; 10.83]
Monthly per capita household income	0.00	0.01	0.01
	[0.00; 0.00]	[0.01; 0.01]	[0.00; 0.01]
Region (0 = North and Northeast; 1 = Mid-South)	0.00	6.31	5.33
	[−0.08; 0.08]	[5.37; 7.25]	[4.50; 6.15]
Area (0 = rural; 1 = urban)	0.11	1.82	1.80
	[0.01; 0.21]	[0.72; 2.93]	[0.83; 2.77]

^a^Includes individuals of all age groups and excluding pregnant women

^b^Classification according to recommendations by the World Health Organization for each age group: BMI above 30 kg/m^2^, for adults and elderly, +3 z-scores, for children under 5 years, and +2 z-scores, for children and adolescents (5 –19 years)

^c^ *n* = 55,970

^d^ *n* = 46,240

## Discussion

Based on data representative of the Brazilian population, 32 % of households had at least one obese individual in the family unit and expenses on medicines were higher in households with obese residents.

No results of studies similar to this one were found, with the household as the analytic unit instead of individuals, and with data on expenses on medicines in the public sector and out-of-pocket expenditures. Our results are consistent with data in studies measuring the influence of obesity on healthcare and medicine expenses in the private or public sector, demonstrating the contribution of obesity in increased expenses on medicines.

A literature review including studies conducted in several countries worldwide estimated that direct medical costs of obese individuals are around 30 % greater than for normal weight subjects (BMI ≤ 25 kg/m^2^) [26]. This difference is higher, but similar to the figure found in this study (19 %) when comparing households with and without obese residents.

A study conducted in Italy in 2001–2002 with adults observed that medicine expenses in the Italian health system for treatment of obese individuals were 153 % greater than for normal weight individuals (BMI 18.5–24.9 kg/m^2^) (335.64 Euros/year × 132.71 Euros/year, respectively) [27].

A reference study for the US presented payer and service-specific estimates for 2008 of annual medical spending attributable to obesity, and found an increase in adult per capita prescription drugs spending attributable to obesity of USD$ 568 (8.3 % increase), for all payers (Medicare, Medicaid and Private) [28]. Prescription expenses of Medicare beneficiaries increased progressively with rising BMI. Individuals that were obese class I (BMI 30–34.9 kg/m^2^), class II (BMI 35–39.9 kg/m^2^) and class III (BMI ≥ 40 kg/m^2^) had spending that was 34, 46 and 69 % higher, respectively, compared to spending of normal weight individuals. After adjusting for confounding variables, the annual mean spending of obese class I individuals was US$ 267 higher than those of normal weight [29]. Another North American study involving individuals aged from 6 to 85 years analysed obesity-related healthcare expenditures with specific analysis of spending on medicines. The results showed that adjusted annual expenditures on medicines among obese females and males were 76 and 59 % higher, respectively, than for normal weight individuals of the same gender [30].

The differences in the magnitude of medicine expenses of obese individuals in comparison to normal weight subjects observed in the studies are due to differences in study perspective, information sources, age and income level of individuals, country of origin as well as healthcare systems characteristics.

Regarding the source of funding for medicines, lower expenses on medicines received in the public sector (SUS and pharmaceutical assistance programs) (around 12 % of total expenses) compared to out-of-pocket expenses were observed. This may be explained by two factors: the investment from the public sector reaches families through freely distributed medicines in health services or co-payment schemes in pharmacies, leading to substantial underestimation by the families of the expenses funded by the public sector; but also by the limitations in access to medicines in the SUS. The co-payment scheme involves payment of the medicines by the Federal Government (90 %) to pharmacies and drug stores from the private network accredited to the “Aqui tem Farmácia Popular” program. These participating pharmacies are rapidly spreading in the country and sell low-cost medications for hypertension, diabetes, cholesterol, asthma, rhinitis, Parkinson’s disease, osteoporosis, glaucoma and birth control [31, 32].

The results of this study are relevant since in developing countries the spending on medicines accounts for 24–66 % of national expenditure on healthcare [33]. In Brazil, household out-of-pocket expenses on medicines were estimated as representing 35 % of healthcare expenses, a percentage substantially higher in lower income families [34]. A study conducted in 2008 involving a representative sample of Brazilian households found that, among individuals using the SUS, prevalence of access to the full range of medicines prescribed was less than 50 % [35]. This finding strengthens the hypothesis that there is still limited access to medicines in the public health system, resulting in higher out-of-pocket spending on health in poorer families, spending which has been characterized as catastrophic due to its negative impact on the family economic situation [36].

The present study used data from household budget surveys and therefore has limitations inherent to this type of data. The information on acquisition of medicines in the HBS refers to the preceding 30 days. Consequently, spending on acute, unstable or seasonal diseases may have been underestimated in the survey. On the other hand, besides being a disease in itself, obesity constitutes a risk factor for a variety of different NCDs [12] for which spending on medicines may be more accurately reported. In addition, there is also the possibility of under-reporting of medicines obtained by individuals, particularly those provided by healthcare services. Due to these limitations, the results of the present study may be underestimated and the actual spending on medicines may be higher.

The questionnaire for collection of data on the acquisition of medicines was filled out by all individuals over the age of 10 residing at the sampled households. However, the intra household distribution of the use of medicines remains unknown, given that a situation may occur where one individual purchases medicines for other members of the household or for children under the age of 10. For this reason and considering that spending decisions are part of family dynamics [17], the household and not the individual, was the study unit and basis for the calculation of average per capita medicine expenses. The influence of factors such as age of the individuals, gender and household income [27, 35, 37, 38], on medicine expenses, were controlled by adjusting for these confounding variables in the analysis. Additional analyses considering the proportions of obese individuals in the household as exposure were carried out, but the results were similar to considering only the presence of one or more obese individuals. The results without discriminating the effect of the the intensity of obesity on household expenses are more straightforward and useful.

## Conclusions

Considering an already unfavourable context, this study revealed that obesity, based on the presence of obese residents in households, is associated with additional medicine expenses, increasing the negative impact on household budgets and public expenditure. Strategies for the prevention of obesity should be strengthened or implemented, along with actions targeting care, treatment and access to medicines of obese individuals. Among other outcomes, these initiatives could reduce the impact of obesity on household expenses on medicines.
